# Multiple Sclerosis Disease Activity and Disability Following Discontinuation of Natalizumab for Pregnancy

**DOI:** 10.1001/jamanetworkopen.2021.44750

**Published:** 2022-01-24

**Authors:** Kerstin Hellwig, Marianne Tokic, Sandra Thiel, Nina Esters, Charlotte Spicher, Nina Timmesfeld, Andrea I. Ciplea, Ralf Gold, Annette Langer-Gould

**Affiliations:** 1 Department of Neurology, St. Josef-Hospital–Katholisches Klinikum Bochum, Ruhr University Bochum, Bochum, Germany; 2 Department of Medical Informatics, Biometry and Epidemiology, Ruhr University Bochum, Bochum, Germany; 3 Department of Neurology, Los Angeles Medical Center, Southern California Permanente Medical Group, Los Angeles, California

## Abstract

**Importance:**

The magnitude of risk of pregnancy-related multiple sclerosis relapses, particularly severe relapses, following natalizumab cessation is unclear, as is whether this risk is reduced by pregnancy or other modifiable factors.

**Objective:**

To determine the association of early natalizumab withdrawal before or during pregnancy with risk of severe relapses and relapse-related disability.

**Design, Setting, and Participants:**

This prospective cohort study used data from the German Multiple Sclerosis and Pregnancy Registry, which enrolled participants between November 2006 and February 2018. Data were collected through structured telephone-administered questionnaires and review of neurologists’ notes. Registry patients who stopped natalizumab treatment within the 2 years before or in the first trimester of pregnancy were included in this analysis. Data were analyzed between January and November 2021.

**Exposures:**

Cessation of natalizumab before pregnancy or until the first trimester.

**Main Outcomes and Measures:**

Severe and significant relapse-related disability was defined as at least a 2.0-point increase on the expanded disability status scale or new or worsening relapse-related ambulatory impairment. Multivariable models accounting for measures of disease severity and repeated events were used.

**Results:**

The cohort comprised 255 women with 274 pregnancies (mean [SD] age at pregnancy onset, 31.25 [4.27] years) who stopped natalizumab before pregnancy (n = 85; median time before last menstrual period, 14.29 weeks [IQR, 3.14-42.43 weeks]) or in the first trimester (n = 189). During pregnancy and the postpartum year, relapses were reported in 183 pregnancies (66.78%), severe relapses in 44 pregnancies (16.05%), and potentially life-threatening relapses in 3 pregnancies (1.10%). One year post partum, significant relapse-related disability was accrued in 29 pregnancies (10.58%). Relapses during pregnancy (n = 109; 39.78%) and in the postpartum period (n = 135; 49.27%) were common. Pregnancy (as a time-dependent covariate) was not associated with a reduced relapse risk (adjusted HR, 0.90; 95% CI, 0.64-1.27). Neither exclusive breastfeeding (adjusted HR, 1.34; 95% CI, 0.86-2.10) nor restarting natalizumab within 4 weeks post partum (adjusted HR, 1.06; 95% CI, 0.48-2.36) were associated with a reduced risk of early postpartum relapses 6 months after delivery. However, the relapse rate ratio during 12 months post partum was lower (0.49; 95% CI, 0.28-0.86) when natalizumab was restarted in the first 4 weeks after birth.

**Conclusions and Relevance:**

This cohort study’s finding suggest that 10% of women may retain clinically meaningful disability from pregnancy-related natalizumab cessation relapses 1 year post partum. This information should be shared with women on natalizumab who desire pregnancy to weigh the high risk of pregnancy-related relapses and disability to the partly uncertain risks of continuing natalizumab throughout pregnancy or switching to depleting agents before conception.

## Key Points

**Question:**

Is early natalizumab cessation before or during the first trimester of pregnancy associated with multiple sclerosis relapse occurrence and relapse-related disability?

**Findings:**

In this cohort study of 274 pregnancies, relapses during pregnancy and postpartum were common, with more than 10% of women retaining clinically meaningful disability from pregnancy-related natalizumab cessation relapses 1 year after delivery. Neither pregnancy itself, restarting natalizumab shortly after delivery, nor exclusive breastfeeding was associated with protection against these relapses.

**Meaning:**

These findings suggest that women with multiple sclerosis need to be informed about the risks of early natalizumab cessation due to pregnancy or planned pregnancy, and treatment alternatives should be considered.

## Teaser

This cohort study examines the associations between cessation of natalizumab therapy in women with multiple sclerosis before or during the first trimester of pregnancy with relapse occurrence and relapse-related disability.

## Introduction

Natalizumab is a highly effective and well-tolerated treatment for relapsing-remitting multiple sclerosis (MS), but if treatment is discontinued, clinical disease activity returns in 9% to 80% of patients after 4 to 7 months.^1^ Pregnancy is thought to be an effective natural treatment and is associated with a reduced relapse risk in the third trimester.^2,3^ However, smaller cohort studies show that relapse and disability progression risk during pregnancy and post partum is higher in women who received natalizumab before pregnancy.^4,5,6,7^ Case reports of severe disease reactivation and rebound up to death in the context of pregnancy planning have been reported.^8,9,10,11,12^ These cases are concerning, yet the magnitude of this risk is still unknown.

Understanding how often severe NATALIZUMAB cessation–related relapses occur in the context of pregnancy planning is crucial for informed, shared decision-making discussions. Simply counting any relapse or sustained disability progression, as is typical of MS randomized clinical trials and MS and pregnancy studies,^4,5,6,7^ doesn’t reflect the disabling relapses patients fear, where the most valuable function for MS patients seems to be lower limb function.^13^

The purpose of this study, therefore, was to describe (1) the absolute risk of severe relapses using a patient-centered definition, (2) persistent disability accrual from these relapses, and (3) the absolute risk of relapses during pregnancy and the postpartum year following NATALIZUMAB cessation. We also examined whether these risks were modified by pregnancy itself, timing of natalizumab cessation, exclusive breastfeeding, and/or resuming natalizumab immediately post partum.

## Methods

### Study Design, Setting, and Participants

**Figure 1.**
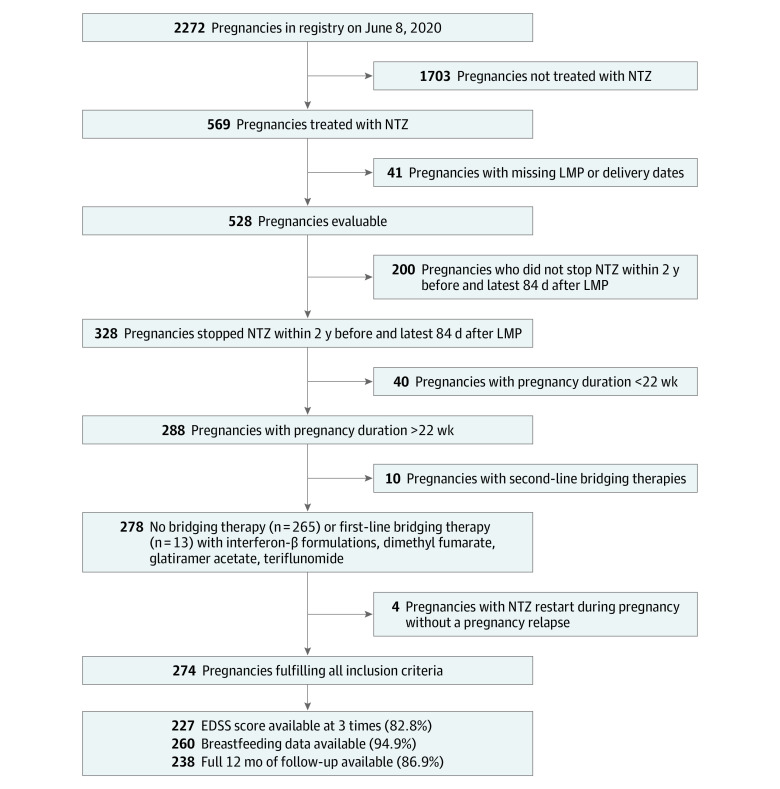
Inclusion Criteria and Eligible Pregnancies in the Cohort Expanded disability status scale (EDSS) values were reported for 227 pregnancies for 3 periods (baseline to 3 months before last menstrual period, third trimester of pregnancy, and postpartum 12 months ±6 weeks, all at least 30 days after a relapse) from the treating neurologists. Data on breastfeeding behavior, including when supplemental feeding was introduced in those breastfeeding exclusively for more than 2 months, was available for 260 pregnancies. LMP indicates last menstrual period; NTZ, natalizumab.

This cohort study used data from the nationwide German MS and Pregnancy Registry. Included patients were documented in the Registry database as of June 8, 2020, had a documented last menstrual period (LMP) and delivery date, stopped natalizumab treatment within 2 years before or at the latest 84 days after the LMP, had a pregnancy duration of at least 22 weeks, and had no second-line bridging therapy (Figure 1). Women voluntary enrolled during pregnancy (at any time point) between November 2006 and February 2018. Data were collected from participants via standardized, telephone-administered questionnaires at enrollment, during each remaining trimester, and at 1, 3, 6, and 12 months post partum.^14^ No data on patient race or ethnicity were collected. Relapses and expanded disability status scale (EDSS) scores were collected and verified through medical records. This study was approved by the institutional review board of Ruhr-University Bochum, and women gave oral informed consent. This cohort study followed the Strengthening the Reporting of Observational Studies in Epidemiology (STROBE) reporting guideline.

### Outcomes

Expanded disability status scale scores were obtained from treating neurologists’ records as follows: before pregnancy (up to 3 months), during the last pregnancy trimester, at 12 months post partum (±7 weeks), and at least 30 days before and after a relapse.^15^ For those with relapses, maximum EDSS score during relapse was obtained when available. Relapses were also confirmed by treating neurologists.

Severe relapses until the end of pregnancy or 1 year post partum were defined as new or worsening ambulatory impairment in order to capture clinical meaningful disability end points. We created the Severe Relapse Disability Composite Score (SRDCS), defined as any relapse during pregnancy or post partum leading to either (1) a change of 2 EDSS points, (2) new ambulatory impairment in those without significant prepregnancy ambulatory impairment (EDSS increase from ≤3.5 to ≥4.0 points), or (3) significant worsening ambulatory impairment in women with at least some pre-existing ambulatory impairment (EDSS increase from ≤5.5 to ≥6.0 points [cane or worse]; 6.0 to ≥6.5 points [walker or worse]; 6.5 to ≥7.0 points [wheelchair or worse]; and 7.0 to ≥8.0 points [bedbound with some arm function or worse]). If EDSS score was missing, relapses were considered nonsevere.

Other outcomes included occurrence of any relapse (yes/no), number of relapses during and after pregnancy, during each trimester of pregnancy, and during the first year post partum. Relapse rates were calculated per trimester, defined from LMP plus 84 days (first trimester), the second trimester lasting for 112 days, and the third trimester ending at delivery.

### Statistical Analysis

Data were analyzed between January and November 2021. Analyses were conducted with R, version 4.0.1 and RStudio, version 1.1.463 (R Foundation) with a 2-sided significance level of α = .05. 95% CIs are reported. Univariate analyses were conducted by Fisher exact test (categorical data) or *t* test (normally distributed data) and Mann-Whitney U test (nonnormal data). If not noted otherwise, *P *values from these tests are reported in the tables. Descriptive statistics are reported as mean (SD) or median (IQR) for continuous data and number/denominator (%) for categorical data.

Annualized relapse rates (ARRs) were estimated by 2 multivariable mixed-effects Poisson regression models. To account for the repeated measures approach, a random patient effect was included. The first model, estimating the association of natalizumab cessation group with the ARRs before, during, and after pregnancy, contained following covariates: natalizumab cessation group (prepregnancy group or first-trimester group), time frame (4 quarters of the year before pregnancy, 3 pregnancy trimesters, or 4 quarters of the postpartum year), age at pregnancy onset, disease duration, and gestational week at entry into the cohort.

The second model examining the association of the timing of natalizumab reintroduction and relapse rates in the postpartum year contained the following covariates: natalizumab cessation group, time frame, timing of natalizumab reintroduction (early [<4 weeks from delivery], later [*≥*4 weeks from delivery], or no natalizumab reintroduction through the first postpartum year), and having had a relapse during pregnancy. The results for both models are provided in eTables 1 and 2 in the Supplement. Annualized relapse rates were compared as relapse rate ratios (RRRs with 95% CIs) extracted from planned linear contrasts and tested for significant difference via a *t* test.

As a sensitivity analysis to the Poisson model, we used Cox proportional hazards regression with the Andersen-Gill extension for recurrent events censored at delivery (t0 = 100 days before natalizumab cessation). Pregnancy status (before pregnancy and trimesters 1-3) was modeled as a time-dependent covariate. The full model result is provided in eTable 3 in the Supplement.

Additionally, we conducted 2 standard Cox proportional hazards regression models. In the first model, t0 was the date of the LMP, with the natalizumab cessation group included as a covariate (eTable 4 in the Supplement). In the second model, t0 was the start of the second trimester of pregnancy, and time since natalizumab cessation in days was included as a covariate (eTable 5 in the Supplement).

Relapses occurring in the first 180 days post partum (t0 = date of delivery) were examined using a standard Cox proportional hazards model. Exclusive breastfeeding was defined as breastfeeding without supplemental feedings for at least 2 months^14^ with timing of natalizumab reintroduction as described above (early, later, or no natalizumab reintroduction).

Multivariable logistic regression models were applied to assess the association between natalizumab cessation group and Severe Relapse Disability Composite Score at the end of pregnancy and the first postpartum year, restricted to the 227 pregnancies with 3 available EDSS values. In all models, we included the following variables: natalizumab cessation group, age at LMP, disease duration, baseline EDSS score, having had a relapse in the year before pregnancy (yes or no), and gestational week at enrollment.

To facilitate the comparison with the existing literature, we conducted sensitivity analyses employing the standard definition of disability progression^4^ (defined as a worsening of at least 1.5 EDSS points if baseline EDSS = 0 points, at least 1 point for baseline EDSS of 1-5.5 points, and 0.5 point for baseline EDSS ≥6.0 points) and an outcome of significant clinical worsening^16^ (at least a 2-point increase in patients with an EDSS score <5.5 points, or an increase of at least 1 point in patients with an EDSS score ≥5.5 points). To assess for selection bias sensitivity, analyses stratified by entry into the registry during the first trimester of pregnancy or later were performed and are presented in eTable 8 in the Supplement.

## Results

We included 274 successful pregnancies in 255 women with a mean (SD) age of 31.25 (4.27) years at the time of conception. All EDSS values were available for 227 pregnancies, and breastfeeding behavior was available for 260 pregnancies (Figure 1). Clinical characteristics of participants stratified by relapse type are shown in Table 1. Only 23 participants had some ambulatory impairment, of whom 2 required a cane or more (Table 1).

**Table 1.** Demographic and Clinical Characteristics of Women With Multiple Sclerosis

Characteristic	No. (%)			
	All (N = 274)	No relapse (n = 91)	No severe relapse (n = 139)^a^	Severe relapse (n = 44)^b^
Age at LMP, mean (SD), y	31.25 (4.27)	31.27 (4.24)	31.33 (4.21)	30.97 (4.63)
Disease duration, median (IQR), y	5.98 (3.90-10.02)	6.45 (4.04-9.61)	5.89 (3.89-9.79)	5.69 (3.74-10.57)
Any relapse in year before pregnancy	96 (35.04)	25 (27.47)	57 (41.01)	14 (31.82)
MS-related disability at baseline (n = 227)^c^				
Missing	47 (17.15)	11 (12.09)	36 (25.90)	0
No disability (EDSS 0-2.0)	95/227 (41.85)	39/80 (48.75)	39/103 (37.86)	17/44 (38.64)
Some disability (EDSS 2.5-3.5)	109/227 (48.02)	31/80 (38.75)	55/103 (53.40)	23/44 (52.27)
Some ambulatory impairment, no assist device (EDSS 4.0-5.5)	21/227 (9.25)	10/80 (12.50)	9/103 (8.74)	2/44 (4.55)
Cane required (EDSS 6.0-6.5)	1/227 (0.44)	0	0	1/44 (2.27)
Wheelchair required (EDSS ≥7.0)	1/227 (0.44)	0	0	1/44 (2.27)
Total duration natalizumab treatment pre-pregnancy, median (IQR), y	2.63 (1.91-3.90)	4.09 (2.88-5.61)	2.48 (1.78-3.50)	2.25 (1.90-2.85)
Any relapse under natalizumab treatment	44 (16.06)	11 (12.09)	25 (17.99)	8 (18.18)
Any prior attempt to stop natalizumab	14 (5.11)	1 (1.10)	9 (6.47)	4 (9.09)
Any relapses with prior stopping attempts	8 (2.92)	1 (1.10)	5 (3.60)	2 (4.55)
Natalizumab discontinuation				
In first trimester	189 (68.98)	67 (73.63)	94 (67.63)	28 (63.64)
Prior to pregnancy	85 (31.02)	24 (26.37)	45 (32.37)	16 (36.36)
Timing of natalizumab discontinuation relative to LMP				
Prepregnancy group: time from stopping natalizumab to LMP, median (IQR), wk	14.29 (3.14-42.43)	10.29 (2.21-43.25)	14.71 (4.43-47.29)	13.86 (2.29-27.89)
First-trimester group: time from LMP to stopping natalizumab, median (IQR), wk	2.71 (1.29-3.57)	2.00 (0.93-3.07)	3.00 (1.43-3.96)	2.57 (1.54-3.61)
Gestational wk at enrollment, median (IQR)	11.71 (7.14-21.14)	10.43 (6.61-18.07)	11.86 (7.00-20.71)	16.00 (9.75-28.86)

Abbreviations: EDSS, expanded disability status scale; LMP, last menstrual period; MS, multiple sclerosis.

^a^ Thirty-six pregnancies with relapses in pregnancy or 1-year post partum and missing EDSS value were categorized as nonsevere. Thirty-three relapses in pregnancy and 43 relapses post partum could not be rated for severity.

^b^ At least 1 severe relapse occurred in 44 pregnancies. Severity of relapse was defined as meeting the severe relapse disability composite score.

^c^ For all disability-related analyses, pregnancies with fewer than 3 EDSS values are counted as missing. The denominator for this subgroup analysis is the number of pregnancies with 3 available EDSS values (n = 227).

**Figure 2.**
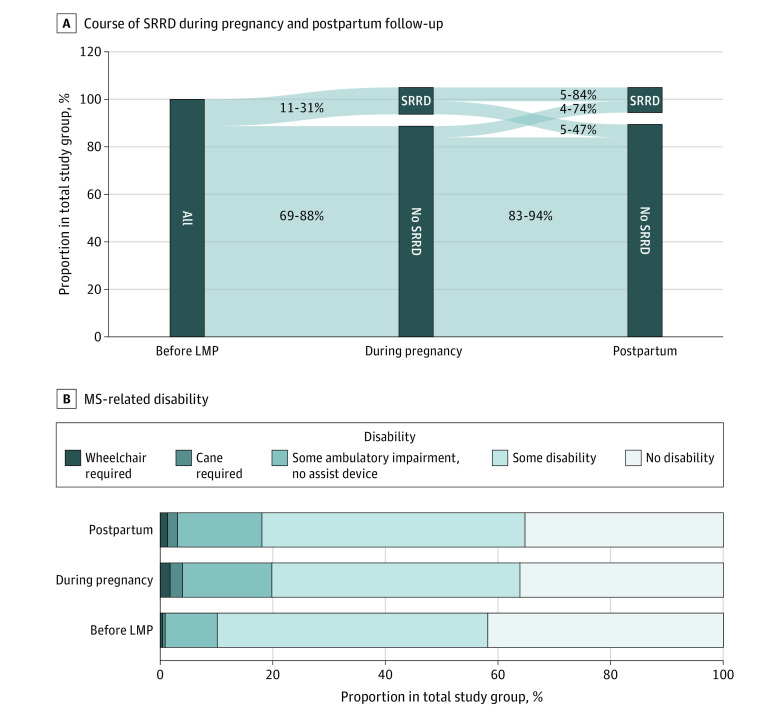
Disability Development During Pregnancy and Post Partum Using the Severe Relapse Disability Composite Score (SRDCS) in 227 Pregnancies With 3 Available Expanded Disability Status Scale (EDSS) Values A, Occurrence of severe relapse-related disability (SRRD) at the end of pregnancy and the first postpartum year. All pregnancies start prior to LMP with no information regarding previous SRRD. The no SRRD category also contains pregnancies with missing data. The denominator of percentages is the total cohort (274 pregnancies). B, Distribution of multiple sclerosis–related disability captured by EDSS before LMP, during pregnancy, and at 1 year post partum in pregnancies with data on multiple sclerosis–related disability. Data on multiple sclerosis–related disability were missing for 47 pregnancies (17.2%). LMP indicates last menstrual period.

During pregnancy and the first postpartum year, we observed relapses in 183 pregnancies (66.78%). At least 1 severe relapse was observed in 44 pregnancies (16.05%). At 1 year post partum, significant relapse-related disability was accrued in 29 pregnancies (10.58%) (Figure 2). During the first postpartum year, 135 relapses (49.27%) were observed.

During pregnancy, at least 1 relapse was reported in 109 pregnancies (39.78%), more than 1 relapse was reported in 42 pregnancies (15.33%) (Table 2). Most relapses (regardless of severity) occurred during the second trimester of pregnancy and in the first 3 months post partum. Severe relapses were more common during pregnancy than post partum. Five women (1.82%) resumed natalizumab during pregnancy owing to a relapse (Table 2).

**Table 2.** Disease Activity During Pregnancy and the Postpartum Period

Variable	No. (%)				*P* value for no relapse vs any relapse	*P* value for severe relapse vs no or nonsevere relapse
	All (N = 274)	No relapse (n = 91)	No severe relapse (n = 139)^a^	Severe relapse (n = 44)^b^		
Pregnancy						
Any relapse in pregnancy	109 (39.8)	0	76 (54.68)	33 (75.00)	NR	NR
More than 1 pregnancy relapse	42 (15.3)	0	22 (15.83)	20 (45.45)	NR	NR
Any relapse in						
1st trimester	24 (8.76)	0	16 (11.51)	8 (18.18)	NR	NR
2nd trimester	75 (27.37)	0	53 (38.13)	22 (50.00)	NR	NR
3rd trimester	44 (16.06)	0	24 (17.27)	20 (45.45)	NR	NR
Any severe relapse in pregnancy^a^	31 (11.31)	0	0	31 (70.45)	NR	NR
Restarted natalizumab in pregnancy	5 (1.82)	0	2 (1.44)	3 (6.82)	.18	.03
Disability during pregnancy (n = 227^c^)						
Information missing	47 (17.2)	11 (12.1)	36 (25.9)	0	NR	NR
Disability progression in pregnancy	40/227 (17.62)	1/80 (1.25)	8/103 (7.77)	31/44 (70.45)	<.001	<.001
Persistent severe relapse related disability in pregnancy	31/227 (13.66)	0	0	31/44 (70.45)	NR	NR
Postpartum period						
Any relapse post partum	135 (49.27)	0	102 (73.38)	33 (75.00)	NR	NR
Lost to follow-up post partum^d^						
Up to 1st trimester	4 (1.46)	3 (3.30)	1 (0.72)	0	NR	NR
Up to 2nd trimester	6 (2.19)	3 (3.30)	3 (2.16)	0	NR	NR
Up to 3rd trimester	13 (4.74)	6 (6.59)	6 (4.32)	1 (2.27)	NR	NR
Up to 4th trimester	36 (13.14)	17 (18.68)	16 (11.51)	3 (6.82)	NR	NR
Timing of any relapse post partum^d^						
Trimester post partum						
1st	86/270 (31.85)	0	65/138 (47.10)	21/44 (47.73)	NR	NR
2nd	48/268 (17.91)	0	36/136 (26.47)	12/44 (27.27)	NR	NR
3rd	35/261 (13.41)	0	29/133 (21.80)	6/44 (13.64)	NR	NR
4th	16/238 (6.72)	0	14/123 (11.38)	2/44 (4.55)	NR	NR
Any severe relapse post partum^a^	15 (5.47)	0	0	15 (34.09)	NR	NR
Disability post partum (n = 227^c^)						
Information missing	47 (17.2)	11 (12.1)	36 (25.9)	0	NR	NR
Disability progression post partum	39/227 (17.18)	2/80 (2.50)	9/103 (8.74)	28/44 (63.64)	<.001	<.001
Persistent severe relapse related disability post partum	29/227 (12.78)	0)	1/103 (0.79)	28/44 (63.64)	NR	NR
Breastfeeding (n = 260^e^)						
Information missing	14 (5.11)	7 (7.69)	7 (5.04)	0	NR	NR
Exclusively	81/260 (31.15)	29/84 (34.52)	41/132 (31.06)	11/44 (25.00)	.41	.16
No breastfeeding	103/260 (39.62)	30/84 (35.71)	51/132 (38.64)	22/44 (50.00)	NR	NR
Some, but not exclusively	76 /260(29.23)	25/84 (29.76)	40/132 (30.30)	11/44 (25.00)	NR	NR
Natalizumab restart post partum (n = 252)						
Missing owing to loss to follow-up before natalizumab restart	22 (8.03)	11 (12.09)	9 (6.47)	2 (4.55)	NR	NR
No natalizumab restart in 1 year post partum	99/252 (39.29)	29/80 (36.25)	55/130 (42.31)	15/42 (35.71)	NR	NR
Resumed natalizumab post partum	153/252 (60.71)	51/80 (63.75)	75/130 (57.69)	27/42 (64.29)	>.99	.52
4 wk or later	83/153 (54.25)	36/51 (70.59)	37/75 (49.40)	10/27 (37.04)	.008	.18
0-4 wk	70/153 (45.75)	15/51 (29.41)	38/75 (50.70)	17/27 (62.96)	NR	NR
Postpartum wk of natalizumab restart, mean (SD)	9.37 (12.35)	12.95 (16.01)	8.20 (10.05)	5.83 (8.26)	.03	.03

Abbreviation: NR, not reported.

^a^ Thirty-six pregnancies with relapses in pregnancy or 1 year post partum and missing expanded disability status values were categorized as nonsevere; 33 relapses in pregnancy and 43 relapses post partum could not be rated for severity.

^b^ Severity of relapse was defined as meeting the Severe Relapse Disability Composite Score.

^c^ The denominator for all disability-related analyses is the number of pregnancies with 3 available EDSS values (n = 227).

^d^ Thirty-six pregnancies were lost to follow-up during the first year post partum, 4 during the first, 2 during the second, 7 during the third, and 23 during the fourth trimester. Denominators for this analysis are the numbers of pregnancies with completed follow-up per postpartum trimester (first trimester, 270; second, 268; third, 261; fourth, 238).

^e^ The denominator for this subgroup analysis is the number of pregnancies with available breastfeeding data (n = 260).

Participants who relapsed or had severe relapses differed in their prepregnancy natalizumab use characteristics and timing of natalizumab discontinuation compared with those who did not relapse, or experienced no or only nondisabling relapses. Women who relapsed had a shorter duration of natalizumab treatment before pregnancy (median duration, 2.48 years [IQR, 1.78-3.50 years] for those with nonsevere relapses and 2.25 years [IQR, 1.90-2.85 years] for those with severe relapses, vs 4.09 years [IQR 2.88-5.61 years] for those with no relapse) and more commonly attempted to stop natalizumab therapy before pregnancy (9 participants [6.47%] with nonsevere relapses and 4 patients [9.09%] with severe relapses vs 1 patient [1.10%] with no relapses) (Table 1).

The majority of participants stopped natalizumab early during the first trimester (n = 189, 68.97%), and 85 participants (31.02%) stopped prior to pregnancy (median time before pregnancy, 14.29 weeks [IQR, 3.14-42.43 weeks]) (Table 1). Only 13 participants (4.74%) used a first-line bridging disease-modifying treatment (Figure 1).

Women with severe relapses joined the study later in pregnancy (median gestational age at enrollment, 16.0 weeks [IQR, 9.75-28.90 weeks] vs 11.3 weeks [IQR, 6.64-18.70 weeks]); 13 women (4.74%) joined after a relapse compared with those who were relapse-free or had only nonsevere relapses (*P* = .005). To account for the possibility of a certain sampling bias, we present the outcomes, depending on whether women joined the registry in the first trimester or later in detail in eTable 8 in the Supplement. Versions of Tables 1 and 2 stratified by natalizumab cessation group are presented in eTables 6 and 7 in the Supplement.

### Severe Relapses and Disability Accumulation

Severe relapses occurred in 31 of 274 pregnancies (11.31%) during pregnancy. In 16 of those pregnancies (51.61%), the woman had persistent severe relapse-related disability by 12 months post partum; and in 15 (48.39%), the woman’s condition improved (Figure 2A). In 2 pregnancies with severe relapses in pregnancy, another severe relapse post partum was observed. Post partum, an additional 13 severe relapses were observed, for a total of 15 severe relapses post partum (Table 2, Figure 2A).

These severe relapses during pregnancy or post partum accounted for the majority of disability worsening (Figure 2B). Only 8 pregnancies had EDSS worsening (median increase, 0.5 points; IQR, 0.5-2.0 points) at 12 months post partum compared with before pregnancy independent of a documented relapse. Multiple sclerosis–related disability remained unchanged throughout pregnancy and the first postpartum year in 175 pregnancies (Figure 2B). The risk for severe relapses during pregnancy was not significantly associated with natalizumab cessation group in multivariable logistic regression (OR, 0.58; 95% CI, 0.24-1.39; *P* = .20). Post partum, the first-trimester group was significantly associated with a lower risk for severe relapses (OR, 0.35; 95% CI, 0.15-0.82; *P* = .02). In contrast, sensitivity analyses using traditional definitions of disability progression^16^ (Table 2) and alternative definitions of significant disability progression (EDSS ≥2.0 points, significant clinical worsening^16^) (eTable 8 in the Supplement), showed no significant association.

Details of the 14 most severe relapses (EDSS increase ≥3 points or an absolute EDSS ≥7.5 points) during pregnancy or post partum are described in eTable 9 in the Supplement. Eleven of those relapses (78.57%) occurred during pregnancy, including 2 unusually severe relapses that both led to an EDSS of 9.5 points, with 1 woman requiring mechanical ventilation. Only 1 woman who had already experienced a relapse during pregnancy (EDSS increase from 2.0 to 5.5 points) had a catastrophic relapse post partum (EDSS = 9.5 points during relapse, EDSS = 8.0 points 12 months post partum). These 3 women (1.09%) were rendered bedbound by their relapses, and 2 now live in nursing homes.

### Influence of Timing of Natalizumab Withdrawal

**Figure 3.**
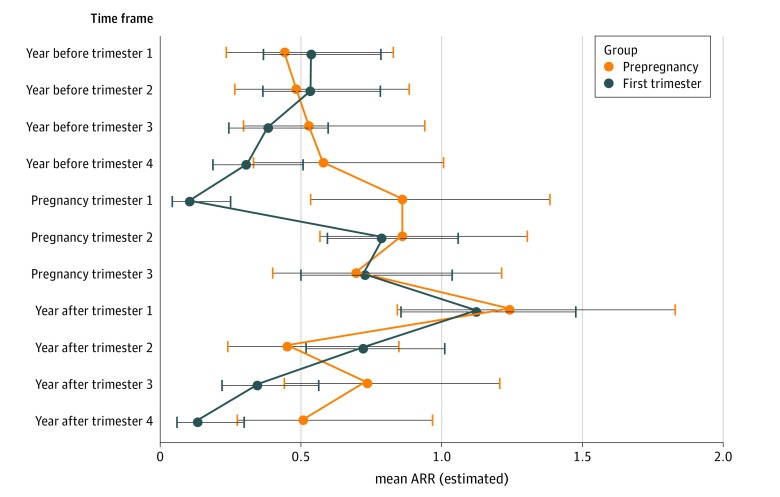
Mean Annual Relapse Rate as Estimated by Zero-Inflated Poisson Regression Stratified by Timing of Natalizumab Cessation The prepregnancy group includes with the last natalizumab infusion before pregnancy but within 2 years of conception. The first-trimester group includes pregnancies with the last natalizumab infusion within the first trimester of pregnancy. Error bars represent 95% CIs. ARR indicates annualized relapse rate.

Among those discontinuing natalizumab before pregnancy, 41 of 85 (48.24%) had at least 1 relapse during pregnancy compared with 68 of 189 (35.98%) who discontinued natalizumab during the first trimester. Women in the first-trimester group had a significantly lower relapse risk during the entire pregnancy than those in the prepregnancy group (RRR, 8.29; 95% CI, 3.06-22.51; *P* < .001), especially during the first trimester (Figure 3). We did not observe a lower relapse rate during the third trimester (Figure 3). Recurrent event analysis with pregnancy as time-dependent covariate did not show a significant reduction of relapse hazard during any pregnancy trimester compared with prepregnancy (HR, 0.90; 95% CI, 0.64-1.27; *P* = .57).

Those who discontinued natalizumab before pregnancy and remained relapse-free or had only nonsevere relapses did so earlier than those with severe relapses (median gestational age at entry into the cohort: no or nonsevere relapses, 11.50 weeks [IQR, 6.36-18.40 weeks]; severe relapses, 12.6 weeks [IQR, 8.86-28.30 weeks]). Women who discontinued natalizumab after pregnancy onset and remained relapse-free (n = 67) stopped natalizumab earlier in the first trimester compared with those with relapses (median days of natalizumab continuation into first trimester: pregnancies with relapses, 3.00 days [IQR, 1.43-3.96 days]; pregnancies without relapses, 2.00 days [IQR, 0.93-3.07 days]) (Table 1).

### Modifiable Risk Factors of Postpartum Relapses: Restarting Natalizumab, Relapses During Pregnancy and Breastfeeding

Most postpartum relapses occurred during the first 3 months post partum, independent of whether natalizumab was suspended before pregnancy or during the first trimester (RRR, 1.11; 95% CI, 0.69-1.76; *P* = .68). Among 260 pregnancies (94.89%) with information on breastfeeding behavior, the women in 157 cases (60.38%) decided to breastfeed, 81 (31.15%) exclusively for at least 2 months (Table 2); only 4 breastfed while receiving disease-modifying treatments (3 under natalizumab, 1 under interferon-β). The 103 women (39.62%) who did not breastfeed at all resumed DMTs earlier than those who breastfed (median, 18.0 days [IQR, 6.0-32.5 days] vs 104.0 days [IQR, 52.0-214.0 days], respectively; *P* < .001). Most women who did not breastfeed restarted natalizumab (78 of 103; 75.73%) or other treatments (10 on fingolimod3 on dimethyl fumarate, 2 on glatiramer acetate, 1 on daclizumab, and 1 on interferon-β). Neither exclusive breastfeeding (adjusted HR, 1.34; 95% CI, 0.86-2.10) (eFigure in the Supplement) nor restarting natalizumab within 4 weeks post partum (adjusted HR, 1.06; 95% CI, 0.48-2.36) were associated with a reduced risk of time to first postpartum relapse 6 months after delivery. However, early natalizumab restart was associated with significantly lower ARR than no natalizumab reintroduction (RRR, 0.49; 95% CI, 0.28-0.9; *P* = .009), but this difference was not statistically significant when comparing early and later natalizumab restart or no and later restart. Relapse rates were similar between the natalizumab cessation groups who restarted natalizumab early (eTable 1 in the Supplement).

## Discussion

In this cohort study, MS relapses during pregnancy or the first postpartum year following natalizumab cessation were exceedingly common. Using a novel patient-centric measure of severe relapses, we found that in 11.31% of women, relapses were so severe that even 1 year post partum they retained meaningful disability, and 1.09% experienced catastrophic relapses to render them newly bedbound. Neither pregnancy itself, restarting natalizumab shortly after delivery, nor exclusive breastfeeding protect against these relapses, but the early postpartum restart of natalizumab reduced the ARR during the entire first postpartum year. The results of our study add relevant information on different maternal risks and may inform a distinct risk-benefit discussion between every neurologist and women on natalizumab who plan a pregnancy.

Single reports of severe disease reactivation after the withdrawal of natalizumab^8,10,11^—some in the context of family planning^9,12^—led us to create a new patient-centered definition of the Severe Relapse Disability Composite Score. Existing MS outcome measures incompletely capture decline in lower limb function, an important outcome for patients,^13^ particularly as a result of incomplete relapse recovery. Thus, existing measures hinder the ability of health care providers to effectively communicate the risk of severe relapse-related disability to patients, particularly in the setting of treatment cessation.

Traditional definitions of sustained disability progression are difficult to explain to patients because they capture a combination of severe relapse-related disability, nonimpairing relapse-related disability, and progression associated with transition to secondary-progressive MS. In this cohort, the traditional definition of sustained disability overestimated the risk of relapse-related disability by 26% because it includes 8 additional women with slow, relapse-independent disability progression. Conversely, a recently proposed definition for significant clinical worsening^16^ underestimated the risk of significant relapse-related disability by 21%. That definition misses women with relapses that cause new worsening in ambulation by requiring a 2-step increase in EDSS score even in women for whom a 1- or 0.5-point increase would result in clinically significant walking difficulties (eg, EDSS increase from 3.0 to 4.0, or cane [6.0] to walker [6.5]).

Previous smaller studies of pregnancy-associated disability worsening following natalizumab^4,16^ and/or fingolimod cessation^17^ reported similar^4,17^ or slightly higher (19%)^16^ proportions of women with disability worsening using various definitions, compared with our cohort.

Our findings starkly contrast with the lack of disease reactivation^17,18,19,20^ reported in larger natalizumab withdrawal studies of nonpregnant patients. The most plausible explanation for this discrepancy is that these studies did not report the risk for clinically meaningful relapse-related disability, relying instead on simply counting relapses using ARR,^18,20^ relapse incidence ratio, number of patients with relapse,^18^ MRI disease activity,^17^ and clinical trial populations instead of real-world cohorts with more aggressive disease. Also, these studies compared relapse count and MRI outcomes following cessation with disease activity before initiation of natalizumab. Finally, pregnancy is a special situation in which a drug may be withdrawn from patients for whom this would not be done in other circumstances without a replacement of treatment, as pregnancy is thought to protect from relapses. Few studies have included any EDSS measurement as an outcome,^1,17,20,21,22^ and in these studies, mean EDSS,^17^ median EDSS without a range,^21^ or sustained disability progression was used. Using means requires the assumption of normal distribution and excludes outliers in the upper 2.5%, which is crucial in reporting rare events. Only 1 study reported stable median EDSS and range in 81 participants who interrupted natalizumab treatment.^20^

The risk of any pregnancy relapse in our study was similar to that of smaller studies of natalizumab withdrawal for pregnancy (39.8% vs 29%-36.5%).^4,7^ We observed the highest relapse risk during the second trimester, similar to another study,^6^ whereas others have reported the highest risk during the first trimester.^4,7^ We think the most likely explanation for this difference is the timing of last natalizumab administration, which occurred very early during the first trimester for the majority of our study’s participants, compared with before conception in others.^4,7^ We also found that pregnancy does not reduce the risk of relapse even after accounting for other risk factors. This finding is new, as previous studies did not model pregnancy as a time-dependent covariate.

As in other studies,^4,5,6,7^ we found an increased relapse risk in the first 3 months post partum, and the most important risk factor for postpartum relapse was relapse during pregnancy. Other studies have associated higher prepregnancy disease severity and relapses during pregnancy with an increased risk of early postpartum relapse.^4,5,6,7^ Exclusive breastfeeding neither decreased nor increased the risk of postpartum relapses, even if it meant foregoing early resumption of disease-modifying treatments.

We found that early postpartum resumption of natalizumab did not reduce the risk of relapses in the first 6 months post partum, in line with the known biology of these early relapses in which a decline in circulating CD4+IFN-g–producing cells, typically beginning during late pregnancy, leads to postpartum MS relapses.^23^ Thus, early resumption was associated with a lower relapse rate in the entire postpartum year, as reported elsewhere,^4,6^ although natalizumab shows a very early effect on the development on gadolinium-enhancing lesions.^24^

We were concerned by 3 very severe relapses experienced by 3 women who became newly bedbound, with 1 requiring mechanical ventilation, with the caveat that 1 of these pregnancies was added to the registry after the occurrence of the catastrophic relapse. Case reports of similarly severe relapses following natalizumab or fingolimod cessation exist,^8,9,10,11,12^ but the absolute risk of such relapses has not been reported. Of note, such severe relapses were not found in the PRIMS (Pregnancy in Multiple Sclerosis) study,^2^ where the worst recorded EDSS was 6.0 points post partum and 5.0 points in 1 woman at the end of pregnancy (Sandra Vukusic, personal communication shared via email on November 9, 2018). Whether such severe relapses occur in other contemporary MS and pregnancy cohorts is unknown and deserves further investigation.

### Strengths and Limitations

Strengths of this study include the importance of the research question, the prospective follow-up of pregnant women, the large sample size, and the development of a new, patient-centered measure of severe relapse-related disability.

Our study also has several limitations inherent to registry studies, including reliance on routine medical records for collecting outcomes and potential selection bias. Although we were able to collect EDSS values for nearly 80% of our participants, we did not obtain data on visual or cognitive function, outcomes that, along with ambulatory function, are important to patients.^13^ Expanded disability status scale scores were also not collected in a standardized way as in clinical trials, raising concerns that some of the EDSS changes are due to interrater reliability.^15^ Because women voluntarily joined our registry at any time during pregnancy, potential selection bias toward more aggressive cases being added after the first trimester is a concern, and our study might overestimate the occurrence of severe relapses. To the extent possible, we examined this bias and found that women who joined the registry later in pregnancy did have more severe disease before joining, but postpartum relapse risks and residual disability were similar for women joining the registry before or in early pregnancy.

## Conclusions

In this cohort study, we found that the risk of relapse-related disability following natalizumab cessation for pregnancy was high, and the risk of catastrophic relapses appeared to exceed the risk of natalizumab-associated progressive multifocal leukoencephalopathy. This risk of experiencing catastrophic relapse after cessation of disease-modifying treatment was likely never a clinical challenge until natalizumab and fingolimod were introduced. Future studies should aim to capture and report these outcomes, as clinical research should ultimately improve patient care^25^ and address outcomes most relevant to the patient. The continuation of natalizumab during pregnancy^6,26,27^ or the use of depleting agents both show promising results in first studies. These treatment options should be evaluated with consideration for the best time to stop natalizumab during pregnancy and restart after delivery. Disease activity and drug safety should also be evaluated in women who are breastfeeding while undergoing monoclonal antibody treatment.^28,29,30^

## Notes

Supplement. **eTable 1.** GLMM Poisson model for relapses postpartum in 252 pregnancies with known timing of NTZ-restart **eTable 2.** Results of the GLMM Poisson model from which the contrasts throughout the results are drawn **eTable 3.** Recurrent event analysis using the Andersen-Gill extension of the cox proportional hazards model **eTable 4.** Survival analysis using the cox proportional hazards model of time to first relapse in pregnancy **eTable 5.** Survival analysis using cox proportional hazards model of time to first relapse from 2^nd^ pregnancy trimester **eTable 6.** Demographic and clinical characteristics of women in the prepregnancy and first-trimester group **eTable 7.** Disease activity during pregnancy and the postpartum period in the prepregnancy and first-trimester group **eTable 8.** Relapse risk and expanded disability status scale change during pregnancy and past-partum **eTable 9.** Clinical characteristics of women with EDSS increase ≥3 or absolute EDSS ≥7.5 during pregnancy or the postpartum year **eFigure.** Time to first relapse postpartum split by breastfeeding behavior
