# Supplementary material for: Multiple Sclerosis Disease Activity and Disability Following Discontinuation of Natalizumab for Pregnancy
Source: JAMA Netw Open. 2022 Jan 24;5(1):e2144750. doi: 10.1001/jamanetworkopen.2021.44750 (PMC8787598; doi:10.1001/jamanetworkopen.2021.44750)
Supplement: Supplement. — eTable 1. GLMM Poisson model for relapses postpartum in 252 pregnancies with known timing of NTZ-restart eTable 2. Results of the GLMM Poisson model from which the contrasts throughout the results are drawn eTable 3. Recurrent event analysis using the Andersen-Gill extension of the cox proportional hazards model eTable 4. Survival analysis using the cox proportional hazards model of time to first relapse in pregnancy eTable 5. Survival analysis using cox proportional hazards model of time to first relapse from 2nd pregnancy trimester eTable 6. Demographic and clinical characteristics of women in the prepregnancy and first-trimester group eTable 7. Disease activity during pregnancy and the postpartum period in the prepregnancy and first-trimester group eTable 8. Relapse risk and expanded disability status scale change during pregnancy and past-partum eTable 9. Clinical characteristics of women with EDSS increase ≥3 or absolute EDSS ≥7.5 during pregnancy or the postpartum year eFigure. Time to first relapse postpartum split by breastfeeding behavior [file jamanetwopen-e2144750-s001.pdf]

# Supplemental Online Content

Hellwig K, Tokic M, Thiel S, et al. Multiple sclerosis disease activity and disability following discontinuation of natalizumab for pregnancy. *JAMA Netw Open*. 2022;5(1):e2144750. doi:10.1001/jamanetworkopen.2021.44750

**eTable 1.** GLMM Poisson model for relapses postpartum in 252 pregnancies with known timing of NTZ-restart

**eTable 2.** Results of the GLMM Poisson model from which the contrasts throughout the results are drawn

**eTable 3.** Recurrent event analysis using the Andersen-Gill extension of the cox proportional hazards model

**eTable 4.** Survival analysis using the cox proportional hazards model of time to first relapse in pregnancy

**eTable 5**. Survival analysis using cox proportional hazards model of time to first relapse from $2^{nd}$ pregnancy trimester

**eTable 6.** Demographic and clinical characteristics of women in the prepregnancy and first-trimester group

**eTable 7.** Disease activity during pregnancy and the postpartum period in the prepregnancy and first-trimester group

**eTable 8.** Relapse risk and expanded disability status scale change during pregnancy and past-partum

**eTable 9.** Clinical characteristics of women with EDSS increase ≥3 or absolute EDSS ≥7.5 during pregnancy or the postpartum year

**eFigure.** Time to first relapse postpartum split by breastfeeding behavior

This supplemental material has been provided by the authors to give readers additional information about their work.

© 2022 Hellwig K et al. *JAMA Network Open.*

## eTable 1: GLMM Poisson model for relapses postpartum in 252 pregnancies with known timing of NTZ-restart

| | **GLMM Poisson: year postpartum** | | |
|---|---|---|---|
| *Coeffcient* | *Incidence Rate Ratios* | *CI (95%)* | *P-Value* |
| Pre-preg.-group | 1.46 | 0.99 – 2.17 | .06 |
| 1st-Tri-group | 1.33 | 0.95 – 1.87 | .10 |
| late NTZ restart | 0.76 | 0.40 – 1.41 | .38 |
| early NTZ restart | 0.42 | 0.19 – 0.93 | **.032** |
| year post. Trim. 2 | 0.60 | 0.42 – 0.87 | **.006** |
| year post. Trim. 3 | 0.45 | 0.30 – 0.67 | **<.001** |
| year post. Trim. 4 | 0.22 | 0.13 – 0.37 | **<.001** |
| Relapse in pregnancy | 1.43 | 1.05 – 1.96 | **.025** |
| late NTZ restart * 1st-Tri-group | 0.91 | 0.43 – 1.95 | .81 |
| early NTZ restart * 1st-Tri-group | 1.40 | 0.56 – 3.55 | .47 |
| **Random Effects** | | | |
| $\sigma^2$ | 1.89 | | |
| $\tau_{00\ ID}$ | 0.00 | | |
| $N_{ID}$ | 252 | | |
| Observations | 990 | | |
| Marginal $R^2$ / Conditional $R^2$ | 0.169 / NA | | |

We analyzed an interaction term of NTZ cessation group and timing of the DMT restart, controlled for having had any relapse in pregnancy. No NTZ restart in one year postpartum was chosen as the reference category to display here.

GLMM, generalized linear mixed model; CI, Confidence Interval; LMP, date of last menstrual period; preg., pregnancy; Pre-preg – group, pregnancies, where NTZ was withdrawn prior to the LMP; 1st-Tri-Group, Pregnancies, where NTZ was withdrawn within the first trimester of pregnancy; NTZ, Natalizumab; year post Trim.1-4, quarter of 91.25 days of postpartum year; No NTZ restart, 99 pregnancies where no NTZ was reintroduced during the postpartum year; late NTZ restart, 83 pregnancies, where NTZ was reintroduced later than 4 weeks postpartum; early NTZ restart, 70 pregnancies, where NTZ was reintroduced up to 4 weeks postpartum

© 2022 Hellwig K et al. *JAMA Network Open.*

**eTable 2: Results of the GLMM Poisson model from which the contrasts throughout the results are drawn**

| | **GLMM Poisson: year prior to pregnancy - year postpartum** | | |
|---|---|---|---|
| *Coeffcient* | *Incidence Rate Ratios* | *CI (95%)* | *P-Value* |
| Pre preg. group | 0.96 | 0.35 – 2.61 | .93 |
| 1stTri-group | 1.16 | 0.49 – 2.76 | .74 |
| year pre. Trim. 2 | 1.10 | 0.47 – 2.58 | .83 |
| year pre. Trim. 3 | 1.20 | 0.52 – 2.77 | .68 |
| year pre. Trim. 4 | 1.31 | 0.57 – 2.99 | .52 |
| preg. Trim. 1 | 1.95 | 0.90 – 4.23 | .09 |
| preg. Trim. 2 | 1.95 | 0.93 – 4.08 | .08 |
| preg. Trim. 3 | 1.58 | 0.69 – 3.60 | .28 |
| year post. Trim. 1 | 2.82 | 1.37 – 5.80 | **.005** |
| year post. Trim. 2 | 1.02 | 0.43 – 2.46 | .96 |
| year post. Trim. 3 | 1.65 | 0.75 – 3.65 | .21 |
| year post. Trim. 4 | 1.17 | 0.49 – 2.81 | .73 |
| Disease duration (years) | 0.99 | 0.96 – 1.01 | .33 |
| Age at LMP (years) | 0.98 | 0.95 – 1.00 | .08 |
| GW at entry into registry | 1.01 | 1.00 – 1.01 | .12 |
| year pre. Trim. 2 * 1stTri-group | 0.91 | 0.33 – 2.48 | .85 |

© 2022 Hellwig K et al. *JAMA Network Open.*

| | | | |
|---|---|---|---|
| year pre. Trim. 3 * 1st Tri-group | 0.60 | 0.22 – 1.65 | .32 |
| year pre. Trim. 4 * 1st Tri-group | 0.44 | 0.16 – 1.23 | .12 |
| preg. Trim. 1 * 1st Tri-group | 0.10 | 0.03 – 0.34 | **<.001** |
| preg. Trim. 2 * 1st Tri-group | 0.76 | 0.32 – 1.81 | .53 |
| preg. Trim. 3 * 1st Tri-group | 0.85 | 0.32 – 2.25 | .75 |
| year post. Trim. 1 * 1st Tri-group | 0.74 | 0.32 – 1.74 | .5 |
| year post. Trim. 2 * 1st Tri-group | 1.32 | 0.48 – 3.61 | .59 |
| year post. Trim. 3 * 1st Tri-group | 0.40 | 0.15 – 1.07 | .07 |
| year post. Trim. 4 * 1st Tri-group | 0.21 | 0.06 – 0.74 | **.015** |
| **Random Effects** | | | |
| $\sigma^2$ | 2.09 | | |
| $\tau_{00\ ID}$ | 0.18 | | |
| ICC | 0.08 | | |
| $N_{ID}$ | 271 | | |
| Observations | 2930 | | |
| Marginal $R^2$ / Conditional $R^2$ | 0.159 / 0.226 | | |

We analyzed an interaction term of NTZ cessation group and timeframe, controlled for disease duration at LMP, age at LMP, and gestational week at entry into the cohort.
GLMM, generalized linear mixed model; CI, Confidence Interval; LMP, date of last menstrual period; preg., pregnancy; Pre preg – group, pregnancies, where NTZ was withdrawn prior to the LMP; 1st-Tri Group, Pregnancies, where NTZ was withdrawn within the first trimester of pregnancy; NTZ, Natalizumab; year pre Trim.1-4, quarter of 91.25 days of the year prior to LMP; preg Trim. 1-3, trimester of pregnancy; year post Trim.1-4, quarter of 91.25 days of postpartum year.

© 2022 Hellwig K et al. *JAMA Network Open.*

## eTable 3: Recurrent event analysis using the Andersen-Gill extension of the cox proportional hazards model

| | **Recurrent Event Analysis: From 100 days prior to NTZ cessation to DOD** | | |
|---|---|---|---|
| *Coeffcient* | *Estimates* | *CI (95%)* | *P-Value* |
| 1st-Tri-group | 0.68 | 0.31 – 1.48 | .36 |
| Trimester1 | 1.21 | 0.73 – 2.02 | .40 |
| Trimester2 | 0.78 | 0.48 – 1.27 | .26 |
| Trimester3 | 0.87 | 0.45 – 1.67 | .66 |
| Age at LMP (years) | 0.96 | 0.93 – 1.00 | **.044** |
| Disease duration at LMP (years) | 1.02 | 0.98 – 1.05 | .33 |
| 1st-Tri-group * Trimester1 | 0.38 | 0.10 – 1.43 | .16 |
| 1st-Tri-group * Trimester2 | 1.14 | 0.45 – 2.88 | .79 |
| 1st-Tri-group * Trimester3 | 1.04 | 0.37 – 2.96 | .94 |
| Observations | 1317 | | |
| $R^2$ Nagelkerke | 0.018 | | |

T0 was chosen to be 100 days prior to NTZ cessation, censoring occurred at the day of the delivery. We modeled a time-dependent covariate for the pregnancy status (prior to pregnancy, trimester 1-3) and controlled for age at LMP and, disease duration at LMP. An interaction term was included for the NTZ cessation group and the pregnancy status.
CI, Confidence Interval; LMP, date of last menstrual period; Pre preg – group, pregnancies, where NTZ was withdrawn prior to the LMP; 1st Tri-Group, Pregnancies, where NTZ was withdrawn within the first trimester of pregnancy; NTZ, Natalizumab; Trimester 1-3, trimester of pregnancy; DOD, day of delivery.

© 2022 Hellwig K et al. *JAMA Network Open.*

## eTable 4: Survival analysis using the cox proportional hazards model of time to first relapse in pregnancy

| | **Survival Analysis: t0= LMP, censored at day of delivery** | | |
|---|---|---|---|
| *Coeffcient* | *Estimates* | *Conf. Int (95%)* | *P-Value* |
| 1st Trim. group | 0.60 | 0.40 – 0.88 | **.009** |
| Observations | 274 | | |
| $R^2$ Nagelkerke | 0.024 | | |

T0 was chosen to be the LMP, censoring occurred at the day of delivery.
CI, Confidence Interval; LMP, date of last menstrual period; Pre preg – group, pregnancies, where NTZ was withdrawn prior to the LMP; 1st Tri-Group, Pregnancies, where NTZ was withdrawn within the first trimester of pregnancy; NTZ, Natalizumab

© 2022 Hellwig K et al. *JAMA Network Open.*

## eTable 5: Survival analysis using cox proportional hazards model of time to first relapse from 2nd pregnancy trimester

| | **Survival Analysis: t0= start of 2nd trimester, censored at day of delivery** | | |
|---|---|---|---|
| *Coeffcient* | *Estimates* | *Conf. Int (95%)* | *P-Value* |
| Time since NTZ cessation (days) | 1.00 | 1.00 – 1.00 | .302 |
| Observations | 274 | | |
| $R^2$ Nagelkerke | 0.005 | | |

T0 was chosen to be the start of the 2nd trimester of pregnancy, censoring occurred at the day of delivery.
CI, Confidence Interval; LMP, date of last menstrual period; Pre preg – group, pregnancies, where NTZ was withdrawn prior to the LMP; 1st Tri-Group, Pregnancies, where NTZ was withdrawn within the first trimester of pregnancy; NTZ, Natalizumab

© 2022 Hellwig K et al. *JAMA Network Open.*

## eTable 6: Demographic and clinical characteristics of women in the prepregnancy and first-trimester group

| | **All**<br>**N= 274** | **Pre-Preg-group**<br>**N=85** | **1st-Tri-group**<br>**N=189** |
|---|---|---|---|
| | n (%) | n (%) | n (%) |
| Age at LMP (years), mean (SD) | 31.25 (4.27) | 31.50 (4.28) | 31.14 (4.28) |
| Disease Duration (years), median [IQR] | 5.98 [3.90;10.02] | 5.97 [3.92;10.15] | 5.98 [3.87;10.01] |
| Any relapse in year prior to pregnancy | 96 (35.04) | 35 (41.18) | 61 (32.28) |
| MS related Disability at baseline (N=227[a]) | | | |
| missing | 47 (17.15) | 28 (32.94) | 19 (10.05) |
| no disability (EDSS 0-2.0) | 95/227 (41.85) | 25/57 (43.86) | 70/170 (41.18) |
| some disability, (EDSS 2.5-3.5) | 109/227 (48.02) | 30/57 (52.63) | 79/170 (46.47) |
| some ambulatory impairment, no assist device (EDSS 4.0-5.5) | 21/227 (9.25) | 2/57 (3.51) | 19/170 (11.18) |
| cane required (EDSS 6.0-6.5) | 1/227 (0.44) | 0/57 (0.00) | 1/170 (0.59) |
| wheelchair required (EDSS >=7.0) | 1/227 (0.44) | 0/57 (0.00) | 1/170 (0.59) |
| Total duration NTZ treatment pre-pregnancy (years), median [IQR] | 2.63 [1.91;3.90] | 2.50 [1.43;3.12] | 3.14 [2.09;4.05] |
| Any relapse under NTZ treatment | 44 (16.06) | 15 (17.65) | 29 (15.34) |
| Any prior attempt to stop NTZ | 14 (5.11) | 3 (3.53) | 11 (5.82) |
| Any relapses with prior stopping attempts | 8 (2.92) | 2 (2.35) | 6 (3.17) |
| Gestational week at enrollment (weeks), median [IQR] | 11.71 [7.14;21.14] | 11.79 [6.43;21.14] | 11.57 [7.57;21.14] |

Pre-Preg-group, pregnancies with last NTZ infusion prior to LMP but within 2 years of conception; 1stTri-group, pregnancies with last NTZ infusion within 1st trimester of pregnancy; LMP, last menstrual period; SD, standard deviation; IQR, interquartile range; n, number in group; MS, multiple sclerosis; baseline, within three months prior to conception; EDSS, expanded disability status scale; NTZ, natalizumab; N, denominator.

[a]For all disability related analysis pregnancies with less than 3 EDSS values are counted as missing. Denominator for this subgroup analysis is the number of pregnancies with 3 available EDSS values (N=227).

© 2022 Hellwig K et al. *JAMA Network Open.*

**eTable 7: Disease activity during pregnancy and the postpartum period in the prepregnancy and first-trimester group**

| | **All N= 274** | **Pre-Preg. N=85** | **1st Trim. N=189** | ***P* overall** |
|---|---|---|---|---|
| | n (%) | n (%) | n (%) | |
| **Pregnancy** | | | | |
| Any relapse in pregnancy | 109 (39.78) | 41 (48.24) | 68 (35.98) | .07 |
| More than one pregnancy relapse | 42 (15.33) | 17 (20.00) | 25 (13.23) | .21 |
| Any relapse in 1st trimester | 24 (8.76) | 19 (22.35) | 5 (2.65) | <.001 |
| Any relapse in 2nd trimester | 75 (27.37) | 23 (27.06) | 52 (27.51) | >.99 |
| Any relapse in 3rd trimester | 44 (16.06) | 13 (15.29) | 31 (16.40) | .96 |
| Any severe relapse in pregnancy[a] | 31 (11.31) | 10 (11.76) | 21 (11.11) | <.001 |
| Restarted NTZ in pregnancy | 5 (1.82) | 2 (2.35) | 3 (1.59) | .65 |
| Disability during pregnancy (N=227[b]) | | | | |
| *Information missing* | 47 (17.2) | 28 (32.9) | 19 (10.1) | |
| Disability progression in pregnancy | 40/227 (17.62) | 12/57 (21.05) | 28/170 (16.47) | <.001 |
| Persistent severe Relapse related disability in pregnancy | 31/227 (13.66) | 10/57 (17.54) | 21/170 (12.35) | <.001 |
| **Postpartum period** | | | | |
| Any relapse postpartum | 135 (49.27) | 45 (52.94) | 90 (47.62) | .49 |
| Lost to follow up postpartum[c] | | | | |
| Up to 1st trimester | 4 (1.46) | 1 (1.18) | 3 (1.59) | |
| Up to 2nd trimester | 6 (2.19) | 2 (2.35) | 4 (2.12) | |
| Up to 3rd trimester | 13 (4.74) | 5 (5.88) | 8 (4.23) | |
| Up to 4th trimester | 36 (13.14) | 13 (15.29) | 23 (12.17) | |
| Timing of any relapse postpartum[c] | | | | |
| *1st trimester postpartum* | 86/270 (31.85) | 28/84 (33.34) | 58/186 (31.18) | .91 |
| *2nd trimester postpartum* | 48/268 (17.91) | 10/83 (12.05) | 38/185 (20.54) | .24 |
| *3rd trimester postpartum* | 35/261 (13.41) | 16/80 (20.00) | 19/181 (10.50) | .09 |
| *4th trimester postpartum* | 16/238 (6.72) | 10/72 (13.89) | 6/166 (3.61) | .016 |
| Any severe relapse postpartum[a] | 15 (5.47) | 7 (8.24) | 8 (4.23) | <.001 |
| Disability postpartum (N=227[b]) | | | | |
| *Information missing* | 47 (17.2) | 28 (32.9) | 19 (10.1) | |
| Disability progression postpartum | 39/227 (17.18) | 14/57 (24.56) | 25/170 (14.71) | <.001 |
| Persistent severe Relapse related disability postpartum | 29/227 (12.78) | 12/57 (21.05) | 17/170 (10.00) | <.001 |

© 2022 Hellwig K et al. *JAMA Network Open.*

| | All<br>N= 274 | Pre-Preg.<br>N=85 | 1st Trim.<br>N=189 | *P* overall |
|---|---|---|---|---|
| Breastfeeding (N=260[d]) | | | | |
| *Information missing* | 14 (5.11) | 6 (7.06) | 8 (4.23) | |
| Exclusively | 81/260 (31.15) | 25/79 (31.65) | 56/181 (30.94) | .77 |
| *No breastfeeding* | 103/260 (39.62) | 32/79 (40.51) | 71/181 (39.23) | |
| *Some, but not exclusively* | 76/260 (29.23) | 22/79 (27.85) | 54/181 (29.83) | |
| NTZ restart postpartum (N=252) | | | | |
| *Missing due to LFU before NTZ restart* | 22 (8.03) | 11 (12.94) | 11 (5.82) | |
| *no NTZ restart in one year pp* | 99/252 (39.29) | 40/74 (54.05) | 59/178 (33.15) | |
| Resumed NTZ post-partum | 153/252 (60.71) | 34/74 (45.95) | 119/178 (66.85) | .001 |
| *> 4 weeks/later* | 83/153 (54.25) | 18/34 (52.94) | 65/119 (54.62) | .004 |
| *0-4 weeks/early* | 70/153 (45.75) | 16/34 (47.06) | 54/119 (45.38) | |
| Postpartum *weeks of NTZ restart, mean (SD)* | 9.37 (12.35) | 7.77 (11.39) | 9.82 (12.61) | .37 |

Pre-Preg-group, pregnancies with last NTZ infusion prior to LMP but within 2 years of conception; 1stTri-group, pregnancies with last NTZ infusion within 1st trimester of pregnancy; LMP, last menstrual period; SD, standard deviation; IQR, interquartile range; n, number in group; MS, multiple sclerosis, baseline, within three months prior to conception; EDSS, expanded disability status scale; NTZ, natalizumab; n, number; N, denominator; LFU, lost to follow up

[a]36 pregnancies with relapses in pregnancy or one-year post-partum and missing EDSS value were categorized as "non-severe". 33 relapses in pregnancy and 43 relapses post-partum could not be rated for severity

[b] For all disability related analysis pregnancies with less than 3 EDSS values are counted as missing. Denominator for this subgroup analysis is the number of pregnancies with 3 available EDSS values (n=227)

[c]36 pregnancies are lost to follow up during the first year postpartum, 4 during the first trimester, 2 during the second trimester, 7 during the third trimester and 23 during the fourth trimester. Denominators for this subgroup analysis is the number of pregnancies with completed follow up per postpartum trimester (first trimester N=270, second trimester N=268, third trimester N=261, fourth trimester N=238)

[d]Breastfeeding: Exclusively, pregnancies followed for at least 8 weeks without introduction of supplemental feedings; No breastfeeding, pregnancies without any breastfed meal after delivery; Breastfeeding: Some, but not exclusively, pregnancies with follow up < 8 weeks or with supplemental feeding during the first 2 months. Denominator for this subgroup analysis is the number of pregnancies with available breastfeeding data (n=260)

© 2022 Hellwig K et al. *JAMA Network Open.*

## eTable 8: Relapse risk and expanded disability status scale change during pregnancy and past-partum

| | | **All** | **Joined later in pregnancy** | **Joined in 1st trimester** | ***P* value** |
|---|---|---|---|---|---|
| | | ***N=274*** | ***N=129*** | ***N=145*** | |
| | | ***n (%)*** | ***n (%)*** | ***n (%)*** | |
| | Pregnancies with SRDCS at 3rd trimester | 31 (11.31) | 20 (15.50) | 11 (7.59) | .07 |
| **SRDCS[a]** at 3rd trimester, one year pp and newly at one year pp, n (%) | Pregnancies with SRDCS at one year pp | 29 (10.58) | 18 (13.95) | 11 (7.59) | .16 |
| | Pregnancies newly with SRDCS at one year pp | 13 (4.74) | 6 (4.65) | 7 (4.83) | >.99 |
| **Median (IQR) EDSS** at 3rd trimester and one year pp | EDSS 3rd trimester, median (IQR) | 2.00 [1.00;3.50] | 3.00 [1.00;4.00] | 2.00 [1.00;3.00] | .13 |
| | EDSS at one year pp, median (IQR) | 2.00 [1.00;3.50] | 3.00 [1.00;3.50] | 2.00 [1.00;3.00] | .13 |
| **Median (IQR) change** EDSS at 3rd trimester and one year pp | Delta EDSS between baseline and 3rd trimester, median (IQR) | 0.00 [0.00;0.00] | 0.00 [0.00;0.50] | 0.00 [0.00;0.00] | .09 |
| | Delta EDSS between baseline and one year pp, median (IQR) | 0.00 [0.00;0.50] | 0.00 [0.00;0.50] | 0.00 [0.00;0.38] | .04 |
| | missing | 47 (17.15) | 23 (17.83) | 24 (16.55) | |
| **Significant clinical worsening** (N=227[b]) at 3rd trimester and one year pp, n (%) | Pregnancies with significant clinical worsening at 3rd trimester | 26/227 (11.45) | 16/106 (15.09) | 10/121 (8.26) | .21 |
| | Pregnancies with significant clinical worsening at one year pp | 23/227 (10.13) | 15/106 (14.15) | 8/121 (6.61) | .13 |
| | missing | 47 (17.15) | 23 (17.83) | 24 (16.55) | |
| **Categorical EDSS** (N=227[b]) at 3rd trimester and one year pp, n (%) | Pregnancies with EDSS >= 2 at 3rd trimester | 145/227 (63.88) | 70/106 (66.04) | 75/121 (61.98) | .60 |
| | Pregnancies with EDSS >= 2 at one year pp | 147/227 (64.76) | 72/106 (67.92) | 75/121 (61.98) | .45 |
| | Pregnancies with EDSS >= 3 at 3rd trimester | 96/227 (42.29) | 54/106 (50.94) | 42/121 (34.71) | .03 |
| | Pregnancies with EDSS >= 3 at one year pp | 95/227 (41.85) | 53/106 (50.00) | 42/121 (34.71) | .04 |
| | Pregnancies with EDSS >= 4 at 3rd trimester | 45/227 (19.82) | 27/106 (25.47) | 18/121 (14.88) | .1 |
| | Pregnancies with EDSS >= 4 at one year pp | 41/227 (18.10) | 22/106 (20.75) | 19/121 (15.70) | .48 |

© 2022 Hellwig K et al. *JAMA Network Open.*

| | | All | Joined later in pregnancy | Joined in 1st trimester | *P* value |
|---|---|---|---|---|---|
| | | ***N=274*** | ***N=129*** | ***N=145*** | |
| | | ***n (%)*** | ***n (%)*** | ***n (%)*** | |
| | Pregnancies with EDSS >= 8 at 3rd trimester | 2/227 (0.88) | 1/106 (0.94) | 1/121 (0.83) | .82 |
| | Pregnancies with EDSS >= 8 at one year pp | 2/227 (0.88) | 2/106 (1.89) | 0/121 (0.00) | .28 |
| | missing | 47 (17.15) | 23 (17.83) | 24 (16.55) | |
| **Categorical EDSS change** (N=227[b]) at 3rd trimester and one year pp, n (%) | Pregnancies with delta EDSS >= 2 points between baseline and 3rd trimester | 25/227 (11.01) | 16/106 (15.09) | 9/121 (7.44) | .14 |
| | Pregnancies with delta EDSS >= 2 points between baseline and one year pp | 23/227 (10.13) | 15/106 (14.15) | 8/121 (6.61) | .13 |
| | Pregnancies with delta EDSS >= 3 points between baseline and 3rd trimester | 11/227 (4.85) | 9/106 (8.49) | 2/121 (1.65) | .04 |
| | Pregnancies with delta EDSS >= 3 points between baseline and one year pp | 5/227 (2.20) | 4/106 (3.77) | 1/121 (0.83) | .26 |
| | Pregnancies with delta EDSS >= 4 points between baseline and 3rd trimester | 3/227 (1.32) | 3/106 (2.83) | 0/121 (0.00) | .15 |
| | Pregnancies with delta EDSS >= 4 points between baseline one year pp | 3/227 (1.32) | 2/106 (1.89) | 1/121 (0.83) | .64 |
| | missing | 47 (17.15) | 23 (17.83) | 24 (16.55) | |
| **Disability progression** (N=227[b]) at 3rd trimester and one year pp, n (%) | Pregnancies with disability progression at 3rd trimester | 40/227 (17.62) | 23/106 (21.70) | 17/121 (14.05) | .24 |
| | Pregnancies with disability progression at one year pp | 39/227 (17.18) | 24/106 (22.64) | 15/121 (12.40) | .09 |
| | missing | 47 (17.15) | 23 (17.83) | 24 (16.55) | |
| **Newly reaching EDSS** (N=227[b]) Milestones at 3rd trimester and one year pp, n (%) | Pregnancies with new walking limitation (EDSS >= 4) at 3rd trimester | 22/227 (9.69) | 15/106 (14.15) | 7/121 (5.79) | .08 |
| | Pregnancies with new walking limitation (EDSS >= 4) at one year pp | 9/227 (3.96) | 3/106 (2.83) | 6/121 (4.96) | .62 |
| | Pregnancies newly requiring walking aid (EDSS >= 6 during) at 3rd trimester | 7/227 (3.08) | 5/106 (4.72) | 2/121 (1.65) | .33 |
| | | **All** | **Joined later in pregnancy** | **Joined in 1st trimester** | ***P* value** |

© 2022 Hellwig K et al. *JAMA Network Open.*

| | | N=274 | N=129 | N=145 | |
|---|---|---|---|---|---|
| | | *n (%)* | *n (%)* | *n (%)* | |
| | Pregnancies newly requiring walking aid (EDSS >= 6) at one year pp | 2/227 (0.88) | 1/106 (0.94) | 1/121 (0.83) | .82 |
| | Pregnancies newly bedbound (EDSS >= 8) at 3rd trimester | 2/227 (0.88) | 1/106 (0.94) | 1/121 (0.83) | .82 |
| | Pregnancies newly bedbound (EDSS >= 8) at one year pp | 1/227 (0.44) | 1/106 (0.94) | 0/121 (0.00) | .52 |
| **Relapses** in pregnancy and pp year | Pregnancies with at least one relapse during pregnancy | 109 (39.78) | 59 (45.74) | 50 (34.48) | .08 |
| | Pregnancies with at least one relapse during 1st trimester | 24 (8.76) | 9 (6.98) | 15 (10.34) | .44 |
| | Pregnancies with at least one relapse during 2nd trimester | 75 (27.37) | 43 (33.33) | 32 (22.07) | .05 |
| | Pregnancies with at least one relapse during 3rd trimester | 44 (16.06) | 24 (18.60) | 20 (13.79) | .36 |
| | Number relapses during pregnancy, median (IQR) | 0.00 [0.00;1.00] | 0.00 [0.00;1.00] | 0.00 [0.00;1.00] | .06 |
| | Number relapses during 1st Trim, median (IQR) | 0.00 [0.00;0.00] | 0.00 [0.00;0.00] | 0.00 [0.00;0.00] | .36 |
| | Number relapses during 2nd Trim, median (IQR) | 0.00 [0.00;1.00] | 0.00 [0.00;1.00] | 0.00 [0.00;0.00] | .03 |
| | Number relapses during 3rd Trim, median (IQR) | 0.00 [0.00;0.00] | 0.00 [0.00;0.00] | 0.00 [0.00;0.00] | .27 |
| | Lost to follow up postpartum[c] | | | | |
| | Up to 2nd trimester | 6 (2.19) | 5 (3.88) | 1 (0.69) | |
| | Up to 4th trimester | 36 (13.14) | 15 (11.63) | 21 (14.48) | |
| | Pregnancies with at least one relapse during first 6 months pp (N=268[c]) | 115/268 (42.91) | 62/124 (50.00) | 53/144 (36.81) | .13 |
| | Pregnancies with at least one relapse during first 12 months pp (N=238[c]) | 135/238 (56.72) | 68/114 (59.65) | 67/124 (54.03) | .34 |

Stratified in pregnancies that joined in 1st trimester (median gestation week of enrollment (IQR): 7.3 (1.9 - 11.9)) and in pregnancies that joined later in pregnancy (median gestation week of enrollment (IQR): 21.9 (12.0 – 37.9)). All *P*-values from univariate comparisons. N, denominator; n, number in group; SRDCS, Severe Relapse Disability Composite Score; pp, post-partum; EDSS, Expanded Disability Status Scale; baseline, 3 months prior to conception; IQR, interquartile range; Trim, trimester

[a]36 pregnancies with relapses in pregnancy or one-year postpartum and missing EDSS value were categorized as “non-severe”. 33 relapses in pregnancy and 43 relapses postpartum could not be rated for severity.

[b]For all disability related analysis pregnancies with less than 3 EDSS values are counted as missing. Denominator for this subgroup analysis is the number of pregnancies with 3 available EDSS values (N=227).

[c]36 pregnancies are lost to follow up during the first year postpartum, 4 during the first trimester, 2 during the second trimester, 7 during the third trimester and 23 during the fourth trimester. Denominators for this subgroup analysis is the number of pregnancies with completed follow up per postpartum trimester (first trimester N=270, second trimester N=268, third trimester N=261, fourth trimester N=238)

© 2022 Hellwig K et al. *JAMA Network Open.*

**eTable 9: Clinical characteristics of women with EDSS increase ≥3 or absolute EDSS ≥7.5 during pregnancy or the postpartum year**

| | BASELINE | | | | DURING PREGNANCY | | | | | POSTPARTUM | | | | | | |
|---|---|---|---|---|---|---|---|---|---|---|---|---|---|---|---|---|
| **No** | **Joined in 1st trimester** | **Disease duration, years** | **NTZ exposure duration, days** | **Baseline EDSS** | **No rel in preg** | **Time to first rel after last NTZ, months** | **Worst EDSS and relapse treatment** | **EDSS 3rd trimester** | **Delta EDSS between baseline and 3rd trimester** | **BF** | **DMT resumption** | **Time to pp DMT start, months** | **No rel up to 12 months pp** | **Time to first rel pp after last NTZ, days** | **EDSS at one year pp** | **Delta EDSS between baseline and one year pp** |
| **1** | yes | 13.65 | -16 | 1 | 2 | 5.27<br>7.47 | EDSS unk<br>3.000 mg CS i.v. | 6.5 | 5.5 | no | NTZ | 0 | 1 | 2 | 1 | 0 |
| **2** | no | 7.29 | 17 | 0 | 3 | 3.67<br>6.54<br>7.54 | EDSS 5.5<br>20.000 mg CS i.v.<br>7xIVIG | 5.5 | 5.5 | yes | NTZ | 0.70 | 1 | 271 | 1 | 1 |
| **3** | no | 3.25 | 14 | 0 | 2 | 4.57<br>7.64 | EDSS 5.5<br>10.000 mg CS i.v. | 5.5 | 5.5 | no | NTZ | 0.54 | 0 | | 0 | 0 |
| **4** | no | 1.95 | 31 | 4 | 2 | 3.07<br>4.20 | EDSS 9.5<br>10.000 mg CS i.v.<br>3xPLEX<br>NTZ 300 mg<br>3x10g IVIG<br>mechanical ventilation | 8.5 | 4.5 | no | NTZ | 0.64 | 0 | | 9 | 5 |
| **5** | no | 13.01 | -75 | 3.5 | 2 | 3.00<br>5.04 | EDSS 8.5<br>13.000 mg CS i.v.<br>2x40 mg CS i.th.<br>5xPLEX<br>4xIA<br>5x300 mg NTZ | 7.5 | 4 | no | NTZ | 0.17 | 0 | | 6 | 2.5 |

© 2022 Hellwig K et al. *JAMA Network Open.*

| | | | | | | | | | | | | | | | | |
|---|---|---|---|---|---|---|---|---|---|---|---|---|---|---|---|---|
| | **BASELINE** | | | | **DURING PREGNANCY** | | | | | **POSTPARTUM** | | | | | | |
| **No** | **Joined in 1st trimester** | **Disease duration, years** | **NTZ exposure duration, days** | **Baseline EDSS** | **No rel in preg** | **Time to first rel after last NTZ, months** | **Worst EDSS and relapse treatment** | **EDSS $3^{rd}$ trimester** | **Delta EDSS between baseline and $3^{rd}$ trimester** | **BF** | **DMT resumption** | **Time to pp DMT start, months** | **No rel up to 12 months pp** | **Time to first rel pp after last NTZ, days** | **EDSS at one year pp** | **Delta EDSS between baseline and one year pp** |
| **6** | no | 6.88 | 16 | 2 | 3 | 5<br>6.17<br>7.17 | EDSS 6<br>8.000 mg CS i.v.<br>4x25g IVIG<br>2x60 mg CS i.th. | 6 | 4 | no | NTZ | 0.24 | 1 | 13 | 4 | 2 |
| **7** | no | 11.94 | 23 | 3 | 3 | 3.57<br>5.6<br>6.64 | EDSS unk<br>13.000 mg CS i.v. | 6.5 | 3.5 | no | NTZ | 0.24 | 1 | 118 | 6.5 | 3.5 |
| **8** | no | 3.56 | 21 | 2 | 3 | 3.07<br>5.10<br>6.80 | EDSS 5.5<br>9.000 mg CS i.v.<br>5xIA/PLEX | 5.5 | 3.5 | no | unk | unk | minimum 3 | unk | 9.5/8 | 7.5 |
| **9** | no | 3.43 | -295 | 1.5 | 4 | 12.17<br>14.20 | EDSS 5.5<br>500 mg CS | 5 | 3.5 | no | DAC | 5.24 | 1 | 748 | 3 | 1.5 |
| **10** | yes | 4.45 | 19 | 1 | 1 | 4.54 | EDSS unk<br>3.000 mg CS i.v. | 4 | 3 | yes | NTZ | 5.04 | 2 | 351 | 1 | 0 |
| **11** | no | 7.05 | 17 | 1 | 2 | 3.47<br>4.5 | EDSS unk<br>8.000 mb CS i.v.<br>2x300mg NTZ | 4 | 3 | no | NTZ | 0.27 | 2 | 265 | 1 | 0 |
| **12** | yes | 2.61 | 7 | 2 | 2 | 1.50<br>5.40 | EDSS 7.0<br>8.000 mg CS<br>5xIA | 4.5 | 2.5 | no | NTZ | 7.40 | 2 | 304 | 6.5 | 4.5 |
| | | | | | | | | | | | | | | | | |
| | **BASELINE** | | | | **DURING PREGNANCY** | | | | | **POSTPARTUM** | | | | | | |

© 2022 Hellwig K et al. *JAMA Network Open.*

| No | Joined in 1st trimester | Disease duration, years | NTZ exposure duration, days | Baseline EDSS | No rel in preg | Time to first rel after last NTZ, months | Worst EDSS and relapse treatment | EDSS 3rd trimester | Delta EDSS between baseline and 3rd trimester | BF | DMT resumption | Time to pp DMT start, months | No rel up to 12 months pp | Time to first rel pp after last NTZ, days | EDSS at one year pp | Delta EDSS between baseline and one year pp |
|---|---|---|---|---|---|---|---|---|---|---|---|---|---|---|---|---|
| **13** | yes | 12.76 | 47 | 7.5 | 1 | 2.74 | EDSS 9.5 10.000 mg CS i.v. 3x30 g IVIG 300 mg NTZ | 8.5 | 2 | no | NTZ | 0.74 | 0 | | 7.5 | 0 |
| **14** | no | 16.26 | 7 | 1 | 0 | | | 1 | 0 | yes | NTZ | 2.80 | 1 | 341 | 4 | 3 |

*Worst EDSS during relapse postpartum 9.5 recovered to 8 at the end of the postpartum year.
No, Number; NTZ, natalizumab; baseline, up to 3 months prior to conception; EDSS, expanded disability status scale; rel, relapse; preg, pregnancy; BF, breastfeeding; DMT, disease modifying therapy; pp, postpartum; unk, unkown; mg, milligrams; CS, corticosteroids; i.v., intravenous; IVIG, intravenous immunoglobulins; PLEX, plasma exchange; g, grams; i.th., intrathecal; IA, immunoadsorption; DAC, daclizumab.

© 2022 Hellwig K et al. *JAMA Network Open.*

## eFigure: Time to first relapse postpartum split by breastfeeding behavior

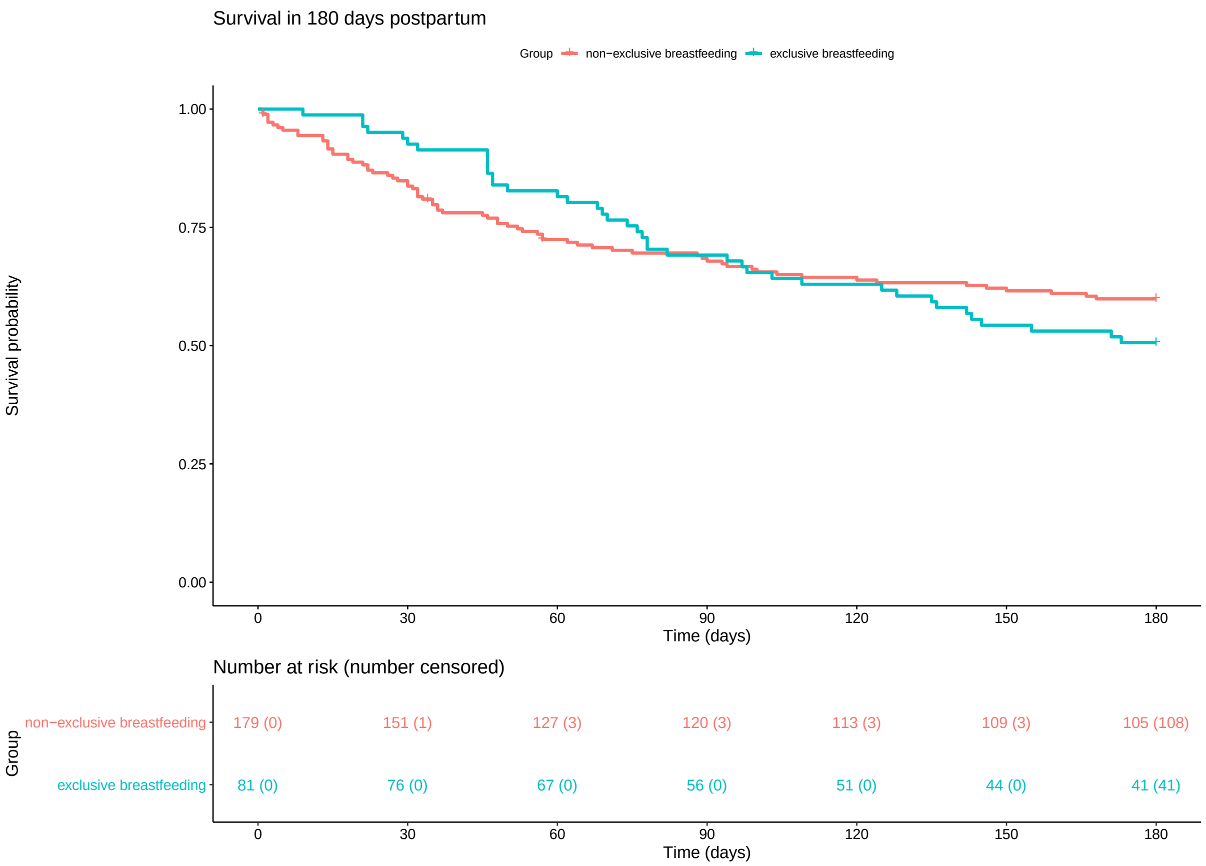


The turquoise curve depicts the survival probability of the exclusive breastfeeding group in the first 180 days postpartum, the red curve the survival probability of the non-exclusive breastfeeding group. The light-colored contours indicate the respective confidence intervals. The red and turquoise numbers depict the number of cases at risk of suffering from a relapse in the respective group at the given timepoints. $t_0$, date of delivery; exclusive breastfeeding, exclusive breastfeeding for at least 8 weeks after delivery with no supplemental feeding; non-exclusive breastfeeding, not breastfeeding or breastfeeding plus formula meals or early reintroduction of disease modifying therapies

© 2022 Hellwig K et al. *JAMA Network Open.*
